# Preterm Birth and Birth Weight and the Risk of Type 1 Diabetes in Chinese Children

**DOI:** 10.3389/fendo.2021.603277

**Published:** 2021-04-14

**Authors:** Ke Huang, Shuting Si, Ruimin Chen, Chunlin Wang, Shaoke Chen, Yan Liang, Hui Yao, Rongxiu Zheng, Fang Liu, Binyan Cao, Zhe Su, Maimaiti Mireguli, Feihong Luo, Pin Li, Hongwei Du, Min Zhu, Yu Yang, Lanwei Cui, Yunxian Yu, Junfen Fu

**Affiliations:** ^1^ Department of Endocrinology, National Clinical Research Center for Child Health, The Children’s Hospital of Zhejiang University School of Medicine, Hangzhou, China; ^2^ Department of Public Health, and Department of Anesthesiology, Second Affiliated Hospital of Zhejiang University School of Medicine, Hangzhou, China; ^3^ Department of Epidemiology & Health Statistics, School of Public Health, School of Medicine, Zhejiang University, Hangzhou, China; ^4^ Department of Endocrinology, Children’s Hospital of Fuzhou, Fuzhou, China; ^5^ Department of Pediatric, The First Affiliated Hospital of Zhejiang University School of Medicine, Hangzhou, China; ^6^ Department of Pediatric, Maternal and Child Health, Hospital of Guangxi Zhuang Autonomous Region, Nanning, China; ^7^ Department of Pediatric, Tongji Hospital, Tongji Medical College, Huazhong University of Science and Technology, Wuhan, China; ^8^ Department of Pediatric, Wuhan Children’s Hospital, Tongji Medical College, Huazhong University of Science & Technology, Wuhan, China; ^9^ Department of Pediatric, Tianjin Medical University General Hospital, Tianjin, China; ^10^ Department of Endocrinology, Zhengzhou Children’s Hospital, Zhenzhou, China; ^11^ Department of Endocrinology, National Medical Center for Children’s Health, Beijing Children’s Hospital, Capital Medical University, Beijing, China; ^12^ Department of Endocrinology, Shenzhen Children’s Hospital, Shenzhen, China; ^13^ Department of Pediatric, The First Affiliated Hospital of Xinjiang Medical University, Urumqi, China; ^14^ Department of Pediatric Endocrinology and Inherited Metabolic Diseases, Children’s Hospital of Fudan University, Shanghai, China; ^15^ Department of Endocrinology, Children’s Hospital of Shanghai Jiaotong University, Shanghai, China; ^16^ Department of Pediatric Endocrinology, The First Bethune Hospital of Jilin University, Changchun, China; ^17^ Department of Endocrinology, Children’s Hospital of Chongqing Medical University, Chongqing, China; ^18^ Department of Endocrinology, Jiangxi Provincial Children’s Hospital, Nanchang, China; ^19^ Department of Pediatric, The First Affiliated Hospital of Harbin Medical University, Harbin, China

**Keywords:** China, preterm birth, birth weight, type 1 diabetes, gender difference

## Abstract

**Aims:**

Findings from previous studies about the association of preterm birth as well as birth weight with the risk of T1DM were still inconsistent. We aimed to further clarify these associations based on Chinese children and explore the role of gender therein.

**Methods:**

A nationwide multicenter and population-based large cross-sectional study was conducted in China from 2017 to 2019. Children aged between 3 and 18 years old with complete information were included in this analysis. Multiple Poisson regression models were used for evaluating the associations of birth weight as well as preterm birth with T1DM in children.

**Results:**

Out of 181,786 children, 82 childhood T1DM cases were identified from questionnaire survey. Children with preterm birth (<37 weeks) had higher risk of type 1 diabetes (OR: 3.17, 95%CI: 1.76-5.71). Children born with high birth weight (≥4,000g) had no statistically significant risk of T1DM (OR:1.71, 95%CI: 0.90-3.22). However, children’s gender might modify the effect of high birth weight on T1DM (girls: OR: 3.15, 95%CI: 1.33-7.47; boys: OR: 0.99, 95%CI: 0.38-2.55, *p* for interaction=0.065). In addition, children with low birth weight were not associated with T1DM (OR: 0.70, 95%CI: 0.24-2.08). The findings from matched data had the similar trend.

**Conclusions:**

In China mainland, preterm birth increased the risk of childhood T1DM, but high birth weight only affected girls. Therefore, early prevention of T1DM may start with prenatal care to avoid adverse birth outcomes and more attention should be paid to children with preterm birth and girls with high birth weight after birth.

## Highlights

What is already known about this subject?

- Some previous studies, including meta-analyses, have indicated that preterm birth and high birth weight had higher risk for childhood type 1 diabetes, but inconsistent results are still emerging.

What is the key question?

- Whether the associations between preterm birth and birth weight and type 1 diabetes based on Chinese children are different for other countries and whether the gender plays a role therein?

What are the new findings?

- Preterm birth was associated with higher risk of childhood T1DM in China.
- High birth weight was only associated with higher risk for T1DM in girls, which had not been reported before.

How might this impact on clinical practice in the foreseeable future?

- Prevention of T1DM may start with prenatal care to avoid adverse birth outcomes.
- More attention should be paid to children with preterm birth and girls with high birth weight after birth to improve the adverse effects.

## Introduction

Type 1 diabetes mellitus (T1DM) is an immune-mediated disease characterized by destruction of pancreatic β-cells, resulting in absolute insulin deficiency (1). The incidence of T1DM is increasing globally with an average annual increase of 3–4% (2). Although, China is at low incidence of T1DM, the incidence among children under 15 years old increased from 0.51 per 100,000 in 1985-1994 to 1.93 per 100,000 in 2010-2013 (3). Human leucocyte antigen (HLA) genotypes is thought to be the major genetic contribution to T1DM but genetic factors fail to completely account for its rapid increase and regional differences under similar genetic background (4–6). In the last 2 decades, there is an increasing clinical interest in birth history. Perinatal factors, including preterm birth and birth weight, have been thought to play an important role in T1DM (2, 7). The estimated rate of preterm birth (gestational age < 37 weeks), low birth weight (<2,500g) and high birth weight (≥4,000g) in China were approximately 6.9%, 4.0% and 7.6%, respectively (8–10). Although previous meta-analyses have shown that preterm birth as well as high birth weight was associated with increased risk of T1DM and low birth weight was not associated with significantly decreased risk of T1DM, several limitations existed as highlighted by authors. For example, lack of consistent adjustment for appropriate confounding factors and most studies were conducted in Europe, America and Australia, which might limit the findings to be extrapolated to population in other countries (11–13). Moreover, inconsistent results are still emerging recently. The latest findings from Swedish cohort of over 4 million people published in 2020 reported that preterm birth (22-36 weeks) was associated with approximately 1.2-fold risk of T1DM among people younger than 18 years (14). While another cross-sectional study in the Middle East indicated that preterm birth was not associated with T1DM during childhood (15). In addition, to our knowledge, a paucity of evidence exists on the association of adverse birth outcomes and childhood T1DM in China and few studies have focused on gender difference. To narrow these gaps, we conducted a population-based cross-sectional study with large sample size in China to determine whether birth weight and preterm birth were significantly associated with T1DM and whether the association could be modified by gender.

## Methods

### Study Design and Population

**Figure 1:**
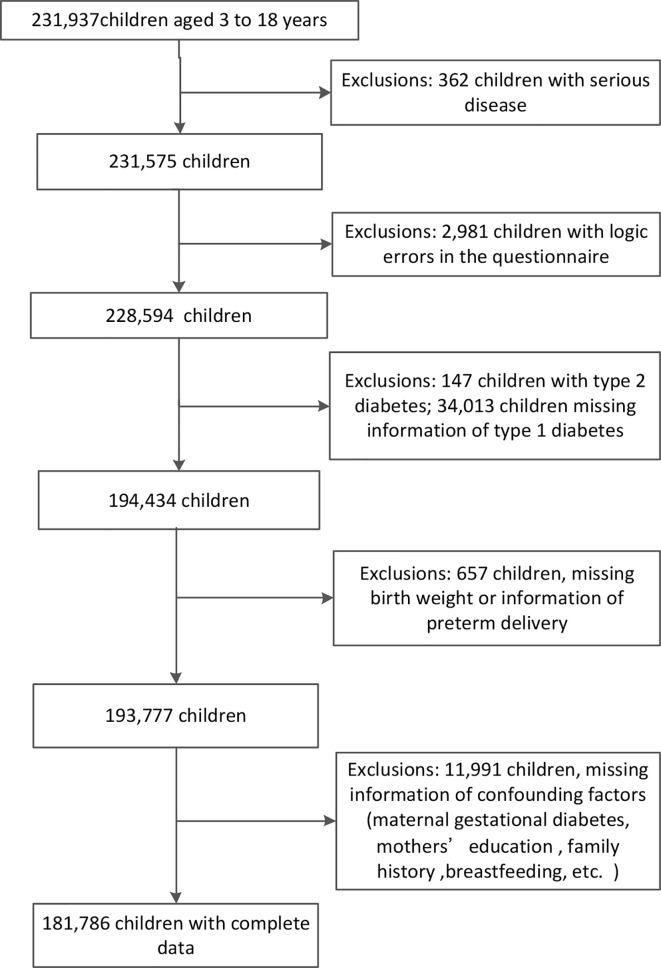
The flowchart of study subjects.

This study was a nationwide large population-based cross-sectional study, conducted at kindergarten, primary, secondary and high schools in 13 medical centers of China (Beijing, Chongqing, Fujian, Guangdong, Guangxi, Henan, Hubei, Jiangxi, Jilin, Shanghai, Tianjin, Xinjiang and Zhejiang) from 2017 to 2019, but Jiangxi was not included in this analysis due to missing the information of T1DM. Schools were selected using stratified cluster random sampling. The study protocol was approved by the ethics board of Zhejiang University. All participants’ informed consents were acquired from children or their parents. 231,937 children aged between 3 and 18 years old were included, and children with serious disease, logic errors in the questionnaire, type 2 diabetes or missing information of key variables were excluded. Finally, 181,786 children were included in this final analysis (**Figure 1**).

### Exposure Variables

Birth weight (g), whether preterm birth or not (according to the definition of <37 gestational weeks, which is the internationally accepted definition of preterm, ICD10 P07.3) were self-reported by parents and children who were born prematurely were further asked about their delivery gestational age. According to the World Health Organization (16), we categorized birth weight into 3 groups: low birth weight (<2,500g), normal birth weight (2,500-3,999g) and high birth weight (≥ 4,000g). Preterm birth was further divided into gestational age of 32th to 37th week and before gestational age of 32th week.

### Outcome Measurement

Childhood T1DM cases were identified by questionnaire surveys with the question: “Has your child been diagnosed with type 1 diabetes? ① Yes ② No.” The questionnaire was completed by their guardians. In addition, to minimize errors, we again asked those who answered yes to confirm the diagnosis of type 1 diabetes.

### Other Variables Definition

The height was measured to the nearest 0.1 cm, whereas weight was measured with a precision of 0.1 kg. The children were weighted without shoes and wearing light clothing. Body mass index (BMI) was calculated as weight (in kg) divided by height (in m) squared. We categorized children’s BMI into four status according to the BMI Z-score (underweight:< -2, normal: -2 to 1, overweight: 1 to 2, and obesity: >2), as defined by WHO 2007 standards and classifications (17). Other variables (demographic characteristics, family history of diabetes, intrauterine exposure and breast feeding) were all acquired from questionnaires. Maternal age at delivery was calculated as maternal age subtracts children’s age at the time of the survey. According the common definition of advanced maternal age (18), we classified into two categories (<35 years old and ≥35 years old).

### Statistical Analysis

We used mean and standard deviations (Mean ± SD) to describe continuous variables, and frequency and percentage (n, %) were reported for categorical variables. Student’s t test and Chi square test were used for continuous variables and categorical variables, respectively, to compare the characteristic difference between groups. Associations of preterm birth as well as birth weight with T1DM were analyzed by multiple Poisson regression, respectively. To explore independent effects, we adjusted potential confounders, including maternal age at delivery, maternal education, annual family income, diabetes of mother, father and siblings, maternal gestational diabetes, children’s characteristics including age, gender and breast feeding. To control the effect of gestational age when analyzed the effects of birth weight on T1DM, whether preterm birth or not was further adjusted. The crossover analysis was conducted to further elucidate the role of preterm birth and birth weight in increasing the risk of T1DM in children. In addition, we also explored the interaction between birth outcomes and gender on childhood T1DM. Interaction analyses with multiplicative interaction terms were conducted. The *P*<0.05 was considered as statistically significant and all statistical analyses were performed using R software (version 3.6.0).

### Sensitivity Analyses

In sensitivity analyses, we repeated the analyses but used the matched data. We identified children with T1DM (n=82) as cases and randomly selected children without T1DM (n=328) as controls on a 1:4 ratio through Propensity Score Matching based on maternal age, maternal education, annual family income, diabetes of mother, father and siblings, maternal GDM, children’s characteristics including age, gender and breast feeding. And conditional logistic regression was used to test the association between preterm birth as well as birth weight and T1DM.

## Results

A total of 181,786 children aged between 3 and 18 years old were included in the analysis (**Figure 1**), among which 82 children were T1DM cases and the prevalence was 45.1 per 100,000. In boys, the prevalence of low birth weight, high birth weight and preterm birth was 3.0%, 11.5% and 5.8%, respectively. In girls, the prevalence of low birth weight, high birth weight and preterm birth was 3.6%, 7.6% and 5.1%, respectively. The comparisons of general characteristics between children with and without T1DM were depicted in **Table 1**. Children with T1DM had a higher proportion of family history (including diabetes of mother, father and siblings), higher proportion of maternal GDM and poor maternal education in comparison with normal children. However, the similar distributions were showed about children’s gender, breastfeeding and obesity, maternal age at delivery and diabetes of grandparents in two groups. All variables were balanced between two groups after Propensity Score Matching (**Supplementary Table 1**).

**Table 1:** Comparison of characteristics between children with and without T1DM.

Variables	T1DM*		*P*	Variables	T1DM		*P*
	No (N=181704)	Yes (N=82)			No (N=181704)	Yes (N=82)	
	n (%)				n (%)		
Maternal age at delivery, years			0.297	Breast feeding duration			0.472
≥35	13370 (7.4)	9 (11.0)		No	37309 (20.5)	21 (25.6)	
Maternal Education			**<0.001**	<6 months	21137 (11.6)	12 (14.6)	
primary school	12993 (7.2)	15 (18.3)		6 to 10 months	60313 (33.2)	23 (28.0)	
junior high school	49299 (27.1)	18 (22.0)		>10 months	62945 (34.6)	26 (31.7)	
senior high school	44927 (24.7)	24 (29.3)		Children’s BMI status			0.839
junior college and above	74485 (41.0)	25 (30.5)		underweight	4931 (2.7)	1 (1.2)	
Annual family income, Ұ			0.086	normal	131343 (72.3)	60 (73.2)	
<100,000	86550 (47.6)	49 (59.8)		overweight	29903 (16.5)	13 (15.9)	
100,000~199,999	54390 (29.9)	18 (22.0)		obesity	15527 (8.5)	8 (9.8)	
≥200,000	40764 (22.4)	15 (18.3)		Birthweight			0.213
Diabetes of mother			**0.003**	<2,500g	5997 (3.3)	4 (4.9)	
Yes	1349 (0.7)	4 (4.9)		2,500~3,999g	158110 (87.0)	66 (80.5)	
Diabetes of father			**0.031**	≥4,000g	17597 (9.7)	12 (14.6)	
Yes	3870 (2.1)	5 (6.1)		Preterm birth			**<0.001**
Diabetes of grandparents			0.470	Yes	9993 (5.5)	14 (17.1)	
Yes	41550 (22.9)	22 (26.8)		Gestational age			**<0.001**
Diabetes of siblings			**0.020**	≥37 weeks	171711 (94.5)	68 (82.9)	
Yes	471 (0.3)	2 (2.4)		32~37 weeks	8896 (4.9)	9 (11.0)	
Maternal GDM^†^			**0.024**	<32 weeks	1097 (0.6)	5 (6.1)	
Yes	7788 (4.3)	8 (9.8)			mean ± SD		
Gender			0.810	Children’s age, years	9.57 ± 3.81	11.79 ± 4.05	**<0.001**
girls	85500 (47.1)	37 (45.1)					

*T1DM, Type 1 diabetes; ^†^GDM, gestational diabetes. Bold values means statistically significant.

### Association of Preterm Birth and Birth Weight With T1DM

As showed in **Table 2**, preterm birth was significantly associated with an increased risk of T1DM after adjusting for or matching potential confounding factors (whole sample size: OR: 3.17, 95%CI: 1.76-5.71; matched sample size: OR: 2.31, 95%CI: 1.14-4.68) and the dose effect emerged when preterm birth was further divided into gestational age of 32th to 37th week (whole sample size: OR: 2.32, 95%CI: 1.14-4.71; matched sample size: OR: 1.68, 95%CI: 0.74-3.81) and before gestational age of 32th (whole sample size: OR: 9.14, 95%CI: 3.63-23.05; matched sample size: OR: 7.01, 95%CI: 1.68-29.78), taking term birth (≥ 37 weeks) as reference. As for birth weight, we initially did not find statistically significant association between children born at neither low birth weight (<2,500g) nor high birth weight (≥4,000g) with childhood T1DM (OR: 0.70, 95% CI: 0.24-2.08; OR: 1.71, 95% CI: 0.90-3.22, respectively). However, high birth weight tended to increase the risk of T1DM in children and then we found that it had significantly higher risk of T1DM (OR: 2.25, 95%CI: 1.03-4.91) when using matched sample size.

**Table 2:** Associations of preterm birth and birth weight with Type 1 diabetes in Chinese children.

Variables	whole sample size				1:4 Matched sample size*			
	n	Type 1 diabetes			n	Type 1 diabetes		
		n (%)	OR (95%CI)	*P*		n (%)	OR (95%CI)	*P*
Preterm birth^†^								
No	171779	68(0.04)	ref.	–	369	68(18.43)	ref.	–
Yes	10007	14(0.14)	**3.17 (1.76-5.71)**	**<0.001**	41	14(34.15)	**2.31 (1.14-4.68)**	**0.021**
Gestational age^†^								
≥37 weeks	171779	68(0.04)	ref.	–	369	68(18.43)	ref.	–
32~36 weeks	8905	9(0.10)	**2.32 (1.14-4.71)**	**0.020**	33	9(27.27)	1.68 (0.74-3.81)	0.211
<32 weeks	1102	5(0.45)	**9.14 (3.63-23.05)**	**<0.001**	8	5(62.50)	**7.01 (1.68-29.78)**	**0.008**
Birth weight^‡^								
<2,500g	6001	4(0.07)	0.70 (0.24-2.08)	0.520	351	66(18.80)	0.38 (0.10-1.46)	0.157
2,500~3,999g	158176	66(0.04)	ref.	–	20	4(20.00)	ref.	–
≥4,000g	17609	12(0.07)	1.71 (0.90-3.22)	0.099	39	12(30.77)	**2.25 (1.03-4.91)**	**0.043**

*Cases were selected according to questionnaire, controls were matched by Propensity Score Matching and the matching variables included maternal age at delivery, maternal education, annual family income, diabetes of mother, father and siblings, maternal gestational diabetes, children’s characteristics including age, gender and breast feeding; Each variable had one model. ^†^Adjustment for variables the same as matching variables above, when using original data; ^‡^Further adjustment for preterm birth or not when using both original and matched data. Bold values means statistically significant.

To further elucidate the independent effect of preterm birth, birth weight on the risk of T1DM in children, we took children with normal birth weight and term birth as reference and found that children with preterm birth but normal birth weight had higher risk of childhood T1DM (OR: 3.61, 95%CI: 1.50-8.67) than children with high birth weight but term birth (OR: 2.24, 95%CI: 1.02-4.89), using the matched sample size. Similar trends were shown when we used original data (**Table 3**).

**Table 3:** The association of birth weight and preterm birth on Type 1 diabetes in Chinese children.

Preterm birth	Birth weight	whole sample size				1:4 Matched sample size*			
		n	Type 1 diabetes			n	Type 1 diabetes		
			n (%)	OR (95%CI)^†^	*P*		n (%)	OR (95%CI)^‡^	*P*
No	2,500~4,000g	151362	56(0.04)	ref.	**-**	328	56(17.07)	ref.	**-**
No	<2,500g	2808	0(0.00)	**-**	**-**	27	0(0.00)	**-**	**-**
No	≥4,000g	17609	12(0.07)	1.68 (0.89-3.16)	0.110	14	12(30.77)	**2.24 (1.02-4.89)**	**0.043**
Yes	2,500~4,000g	6814	10(0.15)	**3.43 (1.72-6.81)**	**<0.001**	23	10(43.48)	**3.61 (1.50-8.67)**	**0.004**
Yes	<2,500g	3193	4(0.13)	**3.24 (1.16-9.03)**	**0.025**	18	4(22.22)	1.45 (0.46-4.41)	0.530

*Cases were selected according to questionnaire, controls were matched by Propensity Score Matching and the matching variables included maternal age at delivery, maternal education, annual family income, diabetes of mother, father and siblings, maternal gestational diabetes, children’s characteristics including age, gender and breast feeding; Each variable had one model. ^†^Adjustment for variables the same as matching variables above. ^‡^Adjustment for nothing. Bold values means statistically significant.

### Gender-Specific Analyses

Considering the possible modification by gender, we conducted analyses stratified by gender. It turned out that findings were different between girls and boys. As shown in **Table 4**, compared to girls born with normal birth weight, girls born with high birth weight had higher risk of T1DM (OR: 3.15, 95%CI: 1.33-7.47), but high birth weight was not associated with T1DM in boys (OR: 0.99, 95%CI: 0.38-2.55). And P for interaction was close to statistically significant (*P* for interaction = 0.065 and 0.069 for adjusted model and matched data, respectively.). However, there was no interaction between gender and preterm birth (*P* for interaction = 0.906 and 0.865 for adjusted model and matched data, respectively. Data was not shown).

**Table 4:** The association between birth weight and Type 1 diabetes stratified by gender in Chinese children.

	n	Type 1 diabetes			n	Type 1 diabetes				*P* for interaction**
		n (%)	OR (95%CI)^†^	*P*		n (%)	OR (95%CI)^‡^		*P*	
			boys				girls			
Birth weight										
Original data										0.065
2,500-3999g	82236	39 (0.05)	ref.	–	75940	27 (0.04)		**ref.**	–	
<2,500g	2918	1 (0.03)	0.28 (0.04-2.22)	0.230	3083	3 (0.10)		1.40 (0.36-5.45)	0.630	
≥4,000g	11095	5 (0.05)	0.99 (0.38-2.55)	0.976	6514	7 (0.11)		**3.15 (1.33-7.47)**	**0.009**	
1:4 Matched data*										0.069
2,500-3,999g	186	39 (20.97)	ref.	–	165	27 (16.36)		**ref.**		
<2,500g	9	1 (11.11)	–	–	11	3 (27.27)		0.99 (0.15-6.62)	0.994	
≥4,000g	23	5 (21.74)	1.23 (0.40-3.80)	0.723	16	7 (43.75)		**8.24 (1.63-41.74)**	**0.011**	

*Cases were selected according to questionnaire, controls were matched by Propensity Score Matching and the matching variables included maternal age at delivery, maternal education, annual family income, diabetes of mother, father and siblings, maternal gestational diabetes, children’s characteristics including age, gender and breast feeding. ^†^Adjustment for variables the same as matching variables above and preterm birth or not; ^‡^Adjustment for preterm birth or not. **P value of the interaction between birth weight and gender. Bold values means statistically significant.

## Discussion

In present national study, both preterm birth and high birth weight were associated with increased risk of T1DM and the effect of preterm birth was stronger than that of high birth weight. However, children’s gender modified the effect of high birth weight on T1DM, with high birth weight only increasing the risk of T1DM in girls.

The adverse effect of preterm birth on T1DM demonstrated in the current study was in line with the findings from previous meta-analysis with 18 studies and 22,073 cases published in 2014 (13) After 2014, three cohort studies in Sweden (14, 19, 20), one cohort study in England (21) and one cohort study in Taiwan (22) also came to similar conclusions. However, one cross-sectional study in Israel indicated that neither early preterm birth (<34 weeks) nor late preterm birth (34-36 weeks) was associated with T1DM during childhood (15). The different results from Israel may be due to different study designs and absence of adjusting for some important confounders, such as family history of diabetes. There are several alternative mechanisms supported the association of preterm birth and T1DM. Firstly, the adverse effects of preterm birth may be related to the accelerator hypothesis (23), which may be plausibly explained by the mechanism that rapid growth increases the demand of insulin secreting and causes β-cell stress and insulin resistance (24, 25). Secondly, permanent changes in insulin sensitivity emerges during the early third trimester (26) and preterm birth may alter development of β-cell mass (27, 28). Thirdly, intrauterine growth restriction is regard as one of mechanisms (29). In addition, preterm birth may also be linked to infection-driven inflammation and gut dysbiosis, which play an important role in the pathophysiology of T1DM (30–32).

When further subdivided gestational age, we found that both children born before 32 and born between 32 and 36 gestational weeks were associated with increased T1DM, furthermore, the dose-response effect was observed. However, results from other studies were different. A population-based register study from Swedish with 14,949 cases found that compared to full-term infants, birth between 32 and 36 gestational weeks had a higher risk (OR: 1.24, 95%CI: 1.14-1.35), while birth before 32 weeks of gestation had a lower risk of childhood-onset T1DM (OR: 0.54, 95%CI: 0.38-0.76) (20). Another national cohort study with 4,193,069 singletons born in Sweden reported increased risk of T1DM among children born at late preterm (34 to 36 gestational weeks) and early term (37 to 38 gestational weeks), while decreased risk among extremely preterm children (22 to 28 gestational weeks) (14). A register-based case-cohort study in Finland reported 21% and 17% increased risk among those born at 33 to 36 gestational weeks and 37 to 38 gestational weeks, respectively but failed to demonstrate decreased risk among birth before 33 gestational weeks (33). We speculate that the difference above might be caused by different divisions of gestational age, different reference levels, study designs and countries.

We found that high birth weight was associated with increased risk of T1DM but low birth weight had no statistically significant effect on T1DM, using the matched sample. It was consistent with one meta-analysis published in 2010 (12). However, inconsistent with our findings, Khashan et al. found no association between high birth weight and T1DM (OR: 1.01, 95%CI: 0.96-1.05) in 2015 (19) and Raphael found a significantly decreased risk of T1DM in children with low birth weight (OR: 0.82, 95%CI: 0.67-0.99) in 2018 (21), which might be explained by different reference levels. The reference levels of birth weight in Khashan’s study (3,000 - 3,999g) and Raphael’s study (3,000- 3,499g) were relatively higher than that in our study (2,500 - 3,999g). Overall, the mechanism behind birth weight and T1DM remains unclear. Kuchlbauer et al. found that there was no sign of excessive weight gain before T1DM among children born at high birth weight (34). Therefore, the effect of high birth weight on T1DM may not be explained by the accelerator hypothesis. Moreover, birth weight might be unlikely to have a direct association with T1DM, and may be a marker of intrauterine exposure, such as maternal nutrition and disease (35). For example, Larsson et al. have demonstrated that general population with high-risk HLA genotypes of T1DM had higher birth weight, but it was due to infections during pregnancy (36). To speculate, the effect of birth weight on T1DM is less direct than that of preterm birth and thus the effect of preterm birth is more obvious and stable.

To our knowledge, only one prior cohort study has reported there was no potential sex-specific difference between preterm birth and T1DM, which was consistent with present study (14). However, we also observed that high birth weight was significantly associated with an increased risk of T1DM among girls while not among boys, which has not been previously reported. The incidence of high birth weight in boys was higher than that in girls but the incidence of T1DM was opposite in China (3). Therefore, we hypothesized that high birth weight had a greater effect on the increased risk of T1DM in girls than in boys. This finding indicated that more attention should be paid to girls with high birth weight after birth for timely detection and treatment of type 1diabetes. Of course, the number of cases in our study was small, which led to poor power (whole sample size: 0.36; matched sample size: 0.68), and thus well-powered studies are warranted to confirm the finding. Whatever, our result may suggest that differences in gender composition may be responsible for inconsistent findings in previous studies and we also provide some information for future research to promote target prevention.

### Strengths and Limitations

There were several strengths in our study. Firstly, to our knowledge, this was the largest study to show the association between birth outcomes and childhood T1DM in China Mainland. Secondly, we considered a wide range of confounders, including demographic characteristics, family history of diabetes, intrauterine exposure and feeding patterns. Thirdly, we also explored the gender differences, which had been less taken into account in previous studies. However, some limitations also should not be ignored. Firstly, although present study was based on large sample size but the number of cases was still relatively small. The power of high birth weight was only 0.36, which might prevent us from finding that the effect of high birth weight was statistically significant. After using propensity score matching, the power increased to 0.68. However, due to the limited number of cases, we were still unable to further divide the gestational weeks into more subgroups. Secondly, in this study, we only investigated the gestational age of children with preterm birth, so it was not possible to assess whether all children had intrauterine growth restriction. However, we adjusted whether preterm birth when exploring the role of birth weight to avoid the effect of preterm birth on birth weight to some extent. Thirdly, cases in this study were from self-report, which might lead to recall bias. But generally, those without disease are not likely to be filled in as sick, so the prevalence rate is likely to be underestimated, and the impact of exposure on the outcome may be underestimated. However, recall bias may also be overestimated, because parents of children with diabetes may be more likely to associate possible factors and remember exposure factors. Finally, we still could not exclude potential confounders such as pregnancy history, pre-pregnancy obesity and pregnancy complications due to the limitations of the investigation and the cross-sectional study failed to consider the effects of recent life styles, which might be influenced by interventions after diagnosis. But previous studies with the adjustment only led to a little alteration, which would have probably made no difference.

In conclusion, children with preterm birth had independently higher risk of childhood T1DM, and girls with high birth weight were more susceptible to have T1DM. Therefore, early prevention should start with prenatal care to avoid adverse birth outcomes and more attention should be paid to children with preterm birth and girls with high birth weight after birth for timely detection and treatment of type 1diabetes.

## Data Availability Statement

The raw data supporting the conclusions of this article will be made available by the authors, without undue reservation.

## Ethics Statement

The study protocol was approved by the Institutional Review Board of The Children’s Hospital of Zhejiang University School of Medicine (Approval Number 2016-JRB-018). Written informed consent to participate in this study was provided by the participants’ legal guardian/next of kin.

## Author Contributions

JF is the chief of the study and is responsible for the study concept and design, funding acquisition and critical revision of the manuscript. KH and SS contributed to analysis and interpreting of the data and writing of the draft. RC, CW, SC, YL, HY, RZ, FL, BC, ZS, MM, FHL, PL, HD, MZ, YY, and LC contributed to study design, investigation and data curation. YXY contributed to the study concept and design, analysis and interpreting of the data and critical revision of the manuscript. All authors contributed to the article and approved the submitted version.

## Funding

This study was funded by Chinese National Natural Science Foundation (81973055), the national key research and development program of China (2016YFC1305301) and the Fundamental Research Funds for the Central Universities. The funder/sponsor did not participate in the work.

## Conflict of Interest

The authors declare that the research was conducted in the absence of any commercial or financial relationships that could be construed as a potential conflict of interest.
