# Supplementary material for: Preterm Birth and Birth Weight and the Risk of Type 1 Diabetes in Chinese Children
Source: Front Endocrinol (Lausanne). 2021 Apr 14;12:603277. doi: 10.3389/fendo.2021.603277 (PMC8079970; doi:10.3389/fendo.2021.603277)
Supplement: Supplementary file 1 [file Table_1.docx]

| Supplementary table 1. Comparison of characteristics between children with and without T1DM, using matched data*. | | | | | | | |
| --- | --- | --- | --- | --- | --- | --- | --- |
| Variables | T1DM† | | *P* | Variables | T1DM | | *P* |
|  | No (N=328) | Yes (N=82) |  |  | No (N=328) | Yes (N=82) |  |
|  | n (%) | |  |  | n (%) | |  |
| Maternal age at delivery, years |  |  | 1.000 | Breast feeding duration |  |  | 0.833 |
| ≥35 | 37 (11.3) | 9 (11.0) |  | No | 81 (24.7) | 21 (25.6) |  |
| Maternal Education | |  | 0.805 | <6 months | 37 (11.3) | 12 (14.6) |  |
| primary school | 52 (15.9) | 15 (18.3) |  | 6 to 10 months | 102 (31.1) | 23 (28.0) |  |
| junior high school | 85 (25.9) | 18 (22.0) |  | >10 months | 108 (32.9) | 26 (31.7) |  |
| senior high school | 102 (31.1) | 24 (29.3) |  | Gender |  |  |  |
| junior college and above | 89 (27.1) | 25 (30.5) |  | girls | 155 (47.3) | 37 (45.1) | 0.824 |
| Annual family income, yuan |  |  | 0.505 | Children's obesity |  |  |  |
| <100,000 | 217 (66.2) | 49 (59.8) |  | Yes | 30 ( 9.1) | 10 (12.2) | 0.533 |
| 100,000~200,000 | 65 (19.8) | 18 (22.0) |  | Birth weight |  |  | 0.208 |
| ≥20,000 | 46 (14.0) | 15 (18.3) |  | <2,500g | 16 ( 4.9) | 4 ( 4.9) |  |
| Diabetes of mother |  |  |  | 2,500~3,999g | 285 (86.9) | 66 (80.5) |  |
| Yes | 26 ( 7.9) | 4 ( 4.9) | 0.477 | ≥4,000g | 27 ( 8.2) | 12 (14.6) |  |
| Diabetes of father |  |  |  | Preterm birth |  |  |  |
| Yes | 23 ( 7.0) | 5 ( 6.1) | 0.961 | Yes | 27 ( 8.2) | 14 (17.1) | **0.029** |
| Diabetes of grandparents | |  |  | Gestational age |  |  | **0.005** |
| Yes | 75 (22.9) | 22 (26.8) | 0.542 | ≥37 weeks | 301 (91.8) | 68 (82.9) |  |
| Diabetes of siblings |  |  |  | 32~37 weeks | 24 ( 7.3) | 9 (11.0) |  |
| Yes | 9 ( 2.7) | 2 ( 2.4) | 1.000 | <32 weeks | 3 ( 0.9) | 5 ( 6.1) |  |
| Maternal GDM ‡ |  |  |  |  | mean ± SD | |  |
| Yes | 34 (10.4) | 8 ( 9.8) | 1.000 | Children's age, years | 11.71 ± 4.07 | 11.79 ± 4.05 | **<0.001** |
| *Cases were selected according to questionnaire, controls were matched by Propensity Score Matching and the matching variables included maternal age, maternal education, annual family income, diabetes of mother, father and siblings, maternal GDM , children's characteristics including age, gender and breast feeding ; †T1DM, Type 1 diabetes; ‡GDM, gestational diabetes | | | | | | | |
